# Health promoting resources and lifestyle factors among higher education students in healthcare and social work programmes: a survey with a longitudinal multicentre design

**DOI:** 10.1186/s12889-024-20506-9

**Published:** 2024-11-08

**Authors:** Aimée Ekman, Sandra Pennbrant, Anders Sterner, Elenita Forsberg, Lena Hedén, Håkan Nunstedt, Annelie J. Sundler, Margaretha Larsson, Ingrid Larsson, Inger Ahlstrand, Hammar Isabelle Andersson, Qarin Lood, Jenny Hallgren

**Affiliations:** 1 School of Health and Welfare, Jönköping University, Box 1026, Jönköping, SE 551 11 Sweden; 2 Department of Health Sciences, University West, Trollhättan, Sweden; 3 Faculty of Caring Science, Work Life and Social Welfare, University of Borås, Borås, Sweden; 4 School of Health and Welfare, Halmstad University, Halmstad, Sweden; 5 School of Health Sciences, University of Skövde, Skövde, Sweden; 6 Department of Health and Rehabilitation, Institute of Neuroscience and Physiology, The Sahlgrenska Academy, University of Gothenburg, Gothenburg, Sweden

**Keywords:** Health, Health promoting resources, Health promoting lifestyle, Students, Health and wellbeing, Higher education, Healthcare, Social work

## Abstract

**Background:**

It has been suggested that the university environment, to improve students’ health status and educational outcomes, should be based on a health promoting approach. More knowledge is needed about health promoting resources and lifestyle factors that may be of value for students in higher education and their future work-life balance. The aim of this study was to explore health-promoting resources, general health and wellbeing, and health promoting lifestyle factors among fourth and final semester students in higher education in healthcare and social work.

**Methods:**

This longitudinal study is based on self-reported data collected through a web-based questionnaire that included questions about general health, wellbeing, and healthy lifestyle factors and made use of instruments: the Sense of Coherence (SOC) scale, the Salutogenic Health Indicator Scale (SHIS), and five questions from the General Nordic Questionnaire (QPS Nordic). The questionnaire was distributed among students enrolled in seven different healthcare and social work programmes at six universities in Sweden. Data was collected when students were in their fourth (2019/2020) and final (2020/2021) semesters analysed with multiple linear and logistic regressions.

**Results:**

The survey included responses from students during the fourth (*n* = 498) and the final (*n* = 343) semester of higher education programmes in health and social work. Total SOC scores decreased between the fourth semester and the final semester. The prevalence of the health promoting lifestyle factor of physical exercise decreased between the fourth and final semesters. Students in their final semester reported valuing group work more highly than did students in their fourth semester. Despite this, students in both the fourth and the final semester reported high SOC, low levels of good general health and perceived wellbeing, and sleeping problems.

**Conclusions:**

Students’ report of good general health were associated with wellbeing, high-intensity physical training, and no sleeping problems A high SOC level was associated with good general health, perceived wellbeing, and no sleeping problems. A higher SHIS level was also associated with wellbeing and no sleeping problems. Therefore, we suggest further research focusing on how to prepare students in healthcare and social work during higher education for a future work-life in balance targeting effects on sleep quality.

**Supplementary Information:**

The online version contains supplementary material available at 10.1186/s12889-024-20506-9.

## Background

Healthy workplaces are important, and the World Health Organization [1] has stressed the need for health promotion to ensure a sustainable working life. If students are to be prepared for future work and professional roles in healthcare and social work and the foundation laid for a sustainable working life [2–4], higher education has an important role to play in strengthening health promoting factors [5]. These consist of individual resources and abilities, such as health behaviours and lifestyle factors [6].

Salutogenesis is an umbrella term for theories that emphasise the causes of health rather than the causes of illness (pathogenesis) and can therefore be a guide for health promotion as action aimed at promoting health as opposed to targeting factors that cause disease [7]. The focus is on understanding health promoting factors and resources that may maintain or improve health on individual, group and organisational levels [8]. The core concept in the salutogenic theory is *sense of coherence* (SOC), or the ability to comprehend the whole of a situation and the capacity to use available resources, including the generalised and specific resistance resources against stress [9, 10]. Resistance resources serve the function of creating conditions enabling people and groups to develop a strong SOC. This strengthens a person’s ability to assess and understand their situation, find meaning, move in a health promoting direction, and manage their situation. SOC encompasses the three dimensions of comprehensibility, manageability and meaningfulness [11]. *Comprehensibility* includes both individual and group-related reflective skills, the understanding of the outside world as structured and orderly, and the ability to comprehend the whole situation. It also covers the capacity to use available resources, and the generalised and specific resistance resources against stress. *Manageability* includes the experience of having the resources to handle various situations. *Meaningfulness* refers to the sense of commitment and motivation to handle different situations [12, 13].

Health promotion and lifestyle factors, especially healthy sleep patterns, are described as elements that can improve students’ health. The processes that the body undergoes during sleep are important for cognitive and physical performance, wellbeing, and quality of life [14–16]. Healthy sleep patterns can also contribute to a well-balanced life and better overall health for students in higher education [17, 18]. There is extensive research focused on physical problems experienced by students in higher education, such as sleep problems and stress [19, 20], and night-time sleep disturbance, which have been shown to predict depression, obsessive-compulsive behaviours, and psychological distress [21, 22]. Some studies show that sleep promotion can effectively improve students’ academic performance [23] and enhance their wellbeing. Research with a dietary focus has shown that unhealthy dietary habits among students in higher education may be associated with higher levels of stress [24] as well as with lower academic performance [25]. The findings of Williams et al. [26] show that a majority of nursing students did not follow food recommendations but had an unhealthy dietary pattern. The study recommends interventions for healthy eating.

Having the support of fellow students, such as through peer support programmes, during studies in higher education can be of great importance for students´ wellbeing [27, 28]. Focusing on the positive aspects of everyday life, including physical activities, can reduce stress-related sleep disorders [29]. Improved quality of life and better mental health are among the reported health benefits of physical activity and exercise [30]. Recently published research [31] shows that students with high levels of healthy lifestyle behaviour also had lower levels of smoking addiction. Although research shows that physical activity and strength training positively affect students’ health and academic performance [16], only a third, or at most two thirds, of students in higher education meet the World Health Organization’s recommendations of approximately 150 min of moderate physical activity per week or at least 60–90 min of high-intensity exercise per week [32–34].

To sum up, awareness of health promoting resources and lifestyle factors may have an impact on students’ health and wellbeing [35–37]. More knowledge is needed about health promoting resources and healthy lifestyle factors that may be of value for students in higher education and for their future work-life balance [38]. It has been suggested that higher education in health-related subjects should favour health promotion strategies [39]. Studies further indicate that health promoting factors are important for a sustainable working life and that such factors may be established or undermined already during the education period [5, 40, 41]. SOC can be health promoting for students in an educational environment [42], as it may improve the health of the individual [43]. Moreover, healthy lifestyle factors and behaviours (e.g. sleep, physical activity, dietary patterns, and smoking) are important determinants of health, wellbeing, and quality of life, as well as of cognitive and physical performance [14–16, 26].

This study reports longitudinal data from the research project “Health promoting factors in higher education for a sustainable working life” [44] and is a follow-up to the baseline study [37]. The aim of this study was to explore health promoting resources, general health and wellbeing and health promoting lifestyle factors among fourth- and final semester students in higher education in healthcare and social work.

Research questions posed:

1. What are the reported levels of health promoting resources and lifestyle factors over time?
2. What is the association of health promoting resources, general health, and healthy lifestyleson reported levels of general health, SOC, and the Salutogenic Health Indicator Scale over time?
3. What is the association of reported levels of health promoting lifestyles, and social study factors on wellbeing over time?

## Methods

The study has a longitudinal design and explores health promoting resources, general health and wellbeing and health promoting lifestyle factors among students in higher education programmes within healthcare and social work at six universities in southern Sweden [44].

A sustainable working life for healthcare and social services professionals is a public health issue of global latitude [1], and in Sweden, sickness absence is high among employees in healthcare and social work [45]. This means that health and welfare students need to be prepared and equipped to be able to handle challenges in their future working life already during their education [4]. Thus, in this study, we have focused on occupations with medium-length healthcare education, i.e. in healthcare and social work, since these groups are very vulnerable when it comes to a sustainable working life.

### Data collection and sample

This study is based on survey data collected via a self-reported, web-based questionnaire (esMaker NX3 software), focusing on general health and wellbeing and lifestyle factors as well as using instruments measuring health promoting resources and processes [37, 44]. At each university, data was collected among students enrolled in the following higher education programmes: biomedical laboratory scientist, diagnostic radiology nurse, occupational therapist, physiotherapist, registered dental hygienist, registered nurse, and social worker. All students who started their higher education studies in one of the included programmes in 2018 received the survey on three occasions. The first occasion was during the first semester, in the spring or autumn of 2018. This baseline data has been analysed elsewhere [37]. The present study is based on data collected among students in their fourth semester in the autumn of 2019 or spring of 2020 and in their final semester (sixth or seventh semester) in the autumn of 2020 or spring of 2021. The same groups of students received the survey halfway through each included semester, and three reminders were sent after that point.

The sample includes 498 students from the fourth semester (438 female gender and 55 male gender) and 343 students from the final semester (312 female gender and 30 male gender). Five students in the fourth semester and one student in the final semester did not identify as either having a female or male gender when answering the survey.

### Measurements

The survey data included questions about demographic characteristics, satisfaction with studies, self-reported general health and wellbeing [37, 44], and health promoting resources as measured by the Sense of Coherence (SOC) scale [9], and the Salutogenic Health Indicator Scale (SHIS) [46]. Questions measuring healthy lifestyles based on the Swedish Public Health Survey of 2018 [47] and items from the General Nordic Questionnaire (QPS Nordic) [47], were also included.

The 13-item SOC scale [9] was employed to examine the participants’ health promoting resources. The SOC scale has high validity and reliability and has been found to be a good indicator of health and psychometric properties [43]. The SOC scale items refer to comprehensibility (five items), manageability (four items), and meaningfulness (four items), and a 7-point semantic scale is used to respond to each item. The total score ranges from 13 to 91, with a higher score indicating a stronger SOC. There is no previous knowledge, as we know, regarding which SOC level that can be regarded as good in relation to young adults. According to Antonovsky [9] SOC still develops in young adults (20–30 years), and experiences of comprehensibility, manageability and meaningfulness can strengthen SOC in these individuals.

The SHIS scale [46] was included in the survey. The SHIS is based on salutogenic theory and holistic descriptions of health and was developed with the support of theories related to the concepts of health and wellbeing [7, 11]. The SHIS is a instrument [46] that includes salutogenic measurements of cognitive, physical, and psychosomatic health. This scale also examines health indicators, intrapersonal characteristics, and interactive functions on a 6-point scale, ranging from negative (scored at 1) to positive (scored at 6). By mistake, the item “having energy” from the SHIS scale was not included in the questionnaire, resulting in 11 items and the analysis was adapted accordingly. The total score ranges from 11 to 66, with a higher score indicating better health.

General health and perceived wellbeing were measured with single questions and dichotomised as excellent/very good/good (1) or less good/bad (0). In addition, eight questions were included from the Swedish Public Health Survey of 2018 [47], assessing healthy lifestyles: high-intensity exercise (dichotomised as yes > 60–90 min/week (1), no < 30–60 min/week (0)); moderate-intensity physical activity, i.e. everyday physical activities, (dichotomised as (yes > 150 min/week (1), no < 150 min/week (0)); being sedentary (yes > 10 h/day (1), no < 10 h/day (0)); sleeping problems (yes (1), no (0)); daily intake of vegetables (yes (1), less than daily intake (0)); consumption of alcohol (yes (1), no or < once per month (0)); smoking (yes (1), seldom/no (0)); daily intake of snuff (oral tobacco) (yes (1), seldom/no (0)).

Five items from the QPS Nordic were used to measure the importance of students’ social support of each other. The QPS Nordic was developed to measure how social support is experienced/perceived in relation to work. For assessment in relation to higher education, questions were selected and modified to fit a student perspective, e.g. substituting “fellow students” and “studies” for “coworkers” and “work”. Each question was answered on a 5-step ordinal scale ranging from “never” (scored at 0) to “often” (scored at 4). High scores indicate strong social interaction.

### Outcome variables

General health, SOC and SHIS were the outcome variables in this study.

### Data analysis

Descriptive analysis was used to describe the participants’ background characteristics. The χ2 test, T-test, and Mann-Whitney U test were used to describe and compare health promoting resources, general health and healthy lifestyles, and social study factors affecting study, in the fourth and final semesters and for differences between participants with male and female genders. Paired Sample T-test or Wilcoxon Signed-Rank Test was used to compare differences on SOC and the included subscales (comprehensibility, manageability, and meaningfulness) and SHIS, in the fourth and final semesters. The Mann-Whitney U-test or Wilcoxon Signed-Rank Test was used if the distribution was not normal according to the Shapiro-Wilk test. Linear and logistic univariate regression was used to test the effect of health promoting resources, general health and healthy lifestyles, and social study factors affecting study on the outcome variables general health, SOC and SHIS, in the fourth and final semesters.

Factors that were significantly associated with general health, SOC and SHIS in the fourth and final semesters, according to the univariate analysis, were simultaneously included in a multiple logistic regression model (general health) and a multiple linear regression model analysis (SOC and SHIS). A *p*-value < 0.05 was considered significant. All data was analysed using SPSS Statistics 27.

## Results

The sample included 498 students from the fourth semester (T4) and 343 students from the final semester (T6/7). Most participants identified themselves as females (T4 88%, T6/7 91%). The participants had a mean age of 28.6 years in the fourth semester and 30.4 years in the final semester. Further descriptions of the participants are provided in Table 1.

**Table 1:** Background characteristics of participants at the fourth and final semester

	T4* Total	Female	Male	T6/7* Total	Female	Male	
	*n* = 498	*n* = 438	*n* = 55	*n* = 343	*n* = 312	*n* = 30	*p*-value**
Age, years, (range 21–61) M (SD)	28.61 (7.31)	28.32 (7.23)	31.07 (7.70)	30.42 (7.67)	30.13 (7.64)	33.40 (7.59)	< 0.001
Gender, % (n)		88.0 (438)	11.0 (55)		91.0 (312)	8.7 (30)	
Residential area (rural) % (n)	23.3 (116)	22.8 (100)	21.8 (12)	20.4 (70)	20.5 (64)	20.0 (6)	0.033

*T4 = fourth semester (2019/2020), T6/7 = sixth or seventh semester (2020/2021)

**Differences between total T4 and total T6/7

### Health promoting resources over time

The mean total SOC score in the fourth semester was 61.43 (*SD* = 11.99), and in the final semester, 61.33 (*SD* = 12.02), a slight but significant decrease (*p* = .032). The mean score for each subscale is presented in Table 2.

**Table 2:** Differences in health promoting resources using SOC subscales and SHIS in the fourth and final semester

	T4 Total	T6/7 Total	T4-T6/7 Change	*p*-value
SOC Total, M (SD)	61.43 (11.99)	61.33 (12.02)	-2.11 (8.86)	**0.032**
Comprehensibility, M (SD)	21.87 (5.59)	21.61 (5.54)	-1.2 (4.22)	**0.010**
Meaningfulness, M (SD)	20.94 (4.13)	21.01 (3.86)	-0.01 (3.75)	0.977
Manageability, M (SD)	18.66 (4.33)	18.76 (4.39)	-0.76 (3.72)	0.061
SHIS, M (SD)	45.24 (9.00)	43.19 (10.87)	-2.05 (10.46)	0.078

SOC = Sense of Coherence Scale, SHIS = The Salutogenic Health Indicator Scale

The mean SHIS score was 45.24 (*SD* = 9.00) in the fourth semester and 43.19 (*SD* = 10.87) in the final semester. However, this decrease was not statistically significant (*p* = .078).

### Health promoting lifestyle factors over time

In total, 88.0% (*n* = 438) students in the fourth semester reported good general health and 84.7% (*n* = 422) reported perceived good wellbeing. In the final semester, there was a non-significant decrease in reported good general health by 86.6% (*n* = 297). However, there was a significant decrease in reported perceived good wellbeing by 79.6% (*n* = 273).

In the fourth semester, 91.4% (*n* = 455) of respondents were non-smokers, and 82.5% (*n* = 411) were not sedentary for more than 10 h per day. Physical exercise (High-intensity exercise > 60–90 min/week) was performed by 58.2% (*n* = 286) of the participants. A total of 79.9% (*n* = 398) were non-users of snuff; 59.8% (*n* = 298) reported low alcohol consumption (never or once a month); and 18.9% (*n* = 94) had a daily intake of vegetables. No sleeping problems were reported by 6.2% (*n* = 31) of the participants in the fourth semester.

In the final semester, 92.7% (*n* = 318) were non-smokers; 81.0% (*n* = 278) did not use snuff daily; and 6.4% (*n* = 22) reported no sleeping problems. One change in the health promoting lifestyle factors between the fourth and the final semester was found to be statistically significant (*p* = .013) in that physical exercise (High-intensity exercise > 60–90 min/week) decreased to 46.4% (*n* = 159).

Perceived good wellbeing as a measurement of a health promoting lifestyle decreased from 84.7% (*n* = 422) in semester four to 79.6% (*n* = 273) in the final semester (*p* = .442). A majority also valued group activities: in semester four, which increased (*p* = .052) in the final semester. The participants’ health-promoting lifestyle factors are presented in Table 3.

**Table 3:** Health promoting lifestyle factors, social study factors, totally, in sex between the fourth and final semester

	Total (T4*) *N* = 498	Female *n* = 438	Male *n* = 55	Total (T6/7*) *n* = 343	Female *n* = 312	Male *n* = 30	*p*-value**
General Health (Good), % (n)	88.0 (438)	88.1 (386)	87.3 (48)	86.6 (297)	86.2 (269)	90.0 (27)	0.369
Perceived wellbeing (Good), (%) n	84.7 (422)	84.7 (371)	87.3 (48)	79.6 (273)	58.7 (183)	36.7 (11)	0.442
No Smoking, (%) n	91.4 (455)	91.3 (400)	92.7 (51)	92.7 (318)	92.9 (290)	90.0 (27)	0.159
No Daily snuff, (%) n	79.9 (398)	81.1 (355)	78.2 (43)	81.0 (278)	83.3 (260)	56.7 (17)	0.417
Consumption of alcohol (Never/once a month), (%) n	59.8 (298)	60.3 (264)	54.5 (30)	55.4 (190)	54.2 (169)	66.7 (20)	0.783
Daily intake of vegetables, Yes, (%) n	18.9 (94)	18.9 (83)	16.4 (9)	22.2 (76)	21.8 (68)	26.7 (8)	0.783
High-intensity exercise > 60–90 min/week, (%) n	58.2 (286)	55.7 (244)	69.1 (38)	46.4 (159)	45.2 (141)	56.7 (17)	**0.013**
Moderate-intensity physical activity > 150 min/week, (%) n	41.8 (205)	40.6 (178)	41.8 (23)	50.4 (173)	50.0 (156)	53.3 (16)	0.483
Sedentary < 10 h/day, (%) n	82.5 (411)	17.1 (75)	80.0 (44)	77.8 (267)	77.6 (242)	83.3 (25)	0.158
No Sleeping problems, (%) n	6.2 (31)	6.4 (28)	5.5 (3)	6.4 (22)	6.7 (21)	3.3 (1)	0.320
Satisfaction with the studies, (%) n	95.0 (473)	95.9 (420)	89.1 (49)	94.8 (325)	94.9 (296)	93.3 (28)	1.00
Support from studymates, Yes, n (%)	77.1 (384)	78.3 (343)	67.3 (37)	76.1 (261)	76.6 (239)	73.3 (22)	0.369
Studymates listening, Yes, n (%)	81.3 (405)	83.1 (364)	67.3 (37)	80.5 (276)	80.8 (252)	80.0 (24)	0.765
Satisfaction with the study choice	95.2 (474)	95.9 (420)	89.1 (49)	95.6 (328)	95.5 (298)	96.7 (29)	0.083
Talk to friends about the studies, Yes, n (%)	83.7 (417)	84.5 (370)	76.4 (42)	84.5 (290)	84.3 (263)	86.7 (26)	0.167
Good relationship with studymates, Yes, n (%)	88.9 (443)	88.8 (389)	89.1 (49)	91.5 (314)	91.0 (284)	96.7 (29)	0.320
Values group work, Yes, n (%)	79.1 (394)	79.9 (350)	72.7 (40)	84.3 (289)	83.7 (261)	90.0 (27)	0.052

*T4 = fourth semester T6/7 = sixth/seventh semester

**Differences between total T4 and total T6/7

### Association between general health and health promoting lifestyle factors

The association between general health and health promoting lifestyle factors was analysed in a univariate analysis model (supplementary files). The significant variables from the univariate analysis model were entered in a multivariate logistic regression model. The fourth semester participants who reported good general health had better perceived wellbeing (*p* = .001) and performed high-intensity physical training > 60–90 min/week (*p* = .001) (Table 4).

**Table 4:** Multiple logistic regression of the predictive factors of good general health in the fourth and final semester

Variables	Dependent variable Good General Health				Variables	Dependent variable Good General Health			
T4*	B (SE)	Odds Ratio	95% CI	*p*-value	T6/7**	B (SE)	Odds Ratio	95% CI	*p*-value
Perceived good wellbeing	2.598 (0.374)	13.443	6.463–14.385	**< 0.001**	Perceived good wellbeing	2.033 (0.393)	7.635	3.535–16.494	**< 0.001**
No Sleeping problems	0.364 (0.573)	0.695	0.226–2.137	0.526	No Sleeping problems	0.110 (0.588)	0.329	0.104–1.044	0.059
High-intensity exercise >60–90 min/week	1.615 (0.373)	5.030	2.423–10.442	**< 0.001**	High-intensity exercise > 60–90 min/week	0.821 (0.412)	2.273	1.013–5.101	**0.046**
Sedentary < 10 h/day	0.356 (0.390)	1.427	0.664–3.067	0.362					
Satisfaction with the studies	0.089 (0.684)	1.094	0.286–4.180	0.286	Satisfaction with the studies	-1.139 (0.705)	0.320	0.080–1.274	0.106
Support from studymates	0.413 (0.520)	1.512	0.546–4.184	0.427	Support from studymates	-0.288 (0.694)	0.750	0.192–2.924	0.750
Studymates listening	-0.654 (0.588)	0.520	0.164–1.645	0.520	Studymates listening	0.321 (0.766)	1.378	0.307–6.185	0.676
Satisfaction with the study choice	0.498 (0.787)	1.645	0.352–7.701	0.527	Satisfaction with the study choice	0.112 (0.818)	0.891	0.225–5.555	0.891
Talk to friends about the studies	0.025 (0.548)	1.025	0.351–2.999	0.963	Talk to friends about the studies	0.304 (0.551)	1.356	0.460–3.993	0.581
Good relationship with studymates	0.521 (0.560)	1.683	0.562–5.042	0.352	Values group work	0.226 (0.487)	1.254	0.483–3.258	0.642

*CI Confidence interval, *R2* = 0.198 (Cox & Snell), σ 0.384 (Nagelkerke), Model χ2 = 108.510, *p* = < 0.001

**CI Confidence interval, *R2* = 0.181 (Cox & Snell), σ 0.329 (Nagelkerke), Model χ2 = 67.005,

*p* = < 0.001

In the final semester, participants who reported good general health perceived betterwellbeing (*p* < .001), performed high-intensity physical training > 60–90 min/week (*p* < .046).

None of the tested social study factors were statistically significantly associated with general health in semester four or the final semester.

### Association between SOC and good general health

The association between the total score of SOC and good general health, respectively, and potential health promoting lifestyle factors was analysed in a univariate linear regression model (SOC) and a univariate logistic regression model (good general health) (supplementary files).

According to the multiple linear regression model for SOC (Table 5), good general health (*p* = .011), perceived good wellbeing (*p* < .001), no sleeping problems (*p* = .020), and had studymates that listen (*p* = .040) in the fourth semester were associated with higher SOC. In the final semester, perceived good wellbeing (*p* < .001), no sleeping problems (*p* = .010), daily intake of vegetables (*p* = .045) and moderate-intensity activity > 150 min/week (*p* = .024) were associated with higher SOC.

**Table 5:** Multiple linear regression analysis of the predictive factors of SOC in the fourth and final semester

Variables	Dependent variable SOC				Variables	Dependent variable SOC			
T4*	β	SE (β)	95% CI	*p*-value	T6/7**	β	SE (β)	95% CI	*p*-value
Good general health	4.375	1.709	1.017–7.733	**0.011**	Good general health	0.606	1.918	-3.168–4.380	0.752
Perceived good wellbeing	11.379	1.600	8.235–14.523	< **0.001**	Perceived good wellbeing	10.670	1.682	7.361–13.980	**< 0.001**
No Sleeping problems	4.995	2.133	0.803–9.187	**0.020**	No Sleeping problems	6.757	2.602	1.637– 11.877	**0.010**
Daily intake of vegetables	-2.026	1.228	-4.440–0.387	0.100	Daily intake of vegetables	-2.825	1.406	-5.591 – -0.058	**0.045**
High-intensity exercise > 60–90 min/week	-0.103	1.014	-2.095–1.889	0.919					
Moderate-intensity activity > 150 min/week	0.888	1.010	-1.097–2.874	0.380	Moderate-intensity activity > 150 min/week	2.695	1.187	0.360–5.029	**0.024**
Studymates listening	3.665	1.777	0.172–7.157	**0.040**	Studymates listening	-0.938	2.507	-5.872–3.995	0.709
Support from studymates	0.333	1.657	-2.924–3.589	0.841	Support from studymates	3.441	2.221	-0.930–7.812	0.122
Sedentary < 10 h/day	1.954	1.296	-0.602–4.492	0.134	Sedentary < 10 h/day	2.020	1.453	-0.838–4.879	0.165
Satisfaction with the study choice	4.138	2.421	-0.610–8.905	. 087	Satisfaction with study choice	3.652	3.225	-2.694–9.998	0.258
Satisfaction with the studies	3.584	2.368	-1.069–8.236	0.131	Satisfaction with the studies	-0.519	3.105	-6.628–5.590	0.867
					Values group work	2.657	1.645	-0.580–5.894	0.107
Talk to friends about the studies	1.374	1.713	-1.993–4.740	0.423	Talk to friends about the studies	3.078	2.107	-1.067–7.224	0.145
Good relationship with studymates	-0.023	1.954	-3.862–3.816	0.991	Good relationship with studymates	-1.479	2.577	-6.550–3.593	0.567

*CI Confidence interval, R2 = 0.286; ANOVA: F = 14.423, df (13, 69); *p* < .001

**CI Confidence interval, R2 = 0.299; ANOVA: F = 10.145, df (13, 310); *p* < .001

### Association between SHIS, health promoting lifestyle factors and social study factors

The association between the total score of SHIS and potential health promoting lifestyle factors and social study factors was analysed in a univariate linear regression model (supplementary files). Factors considered as significant (*p* < .05) from the univariate linear regression model, were entered simultaneously in a multivariable model.

According to the multiple regression model, the total score of SHIS was associated with perceived good wellbeing (*p* < .001) and no sleeping problems (*p* = .018) in the fourth semester. In the final semester, SHIS was associated with perceived good wellbeing (*p* < .001), no sleeping problems (*p* = .013), and satisfaction with the studies (*p* = .014). Please see Table 6.

**Table 6:** Multiple linear regression analysis of the predictive factors of SHIS in the fourth and final semester

Variables	Dependent variable SHIS				Variables	Dependent variable SHIS			
T4*	β	Standard error (β)	95% CI	*p*-value	T6/7**	β	Standard error (β)	95% CI	*p*-value
Good general health	1.925	1.495	-1.012–4.863	0.198	Good general health	1.552	1.601	-1.597–4.702	0.333
Perceived good wellbeing	10.548	1.377	7.842–13.254	**< 0.001**	Perceived wellbeing	9.104	1.366	6.416–11.791	**< 0.001**
No Sleeping problems	4.380	1.845	0.754–8.007	**0.018**	No sleeping problems	5.403	2.158	1.157–9.649	**0.013**
Daily intake of vegetables	-1.764	1.043	-3.813–0.286	0.092					
Sedentary < 10 h/day	1.435	1.093	-0.712–3.582	0.190					
High-intensity exercise > 60–90 min/week	0.947	0.855	-0.733–2.628	0.268	High-intensity exercise > 60–90 min/week	0.762	0.976	-1.159–2.682	0.436
Studymates listening	0.893	1.510	-2.074–3.861	0.554	Studymates listening	-0.395	2.013	-4.356–3.565	0.844
Support from studymates	0.647	1.396	-2.096–3.389	0.643	Support from studymates	1.695	1.766	-1.780–5.170	0.338
Satisfaction with the studies	1.531	1.947	-2.296–5.357	0.432	Satisfaction with the studies	6.132	2.473	1.267–10.998	**0.014**
Values group work	0.682	1.081	-1.442–2.806	0.528	Values group work	-0.853	1.439	-3.684–1.979	0.554
Talk to friends about the studies	1.041	1.509	-1.925–4.007	0.491	Talk to friends about the studies	0.725	1.742	-2.702–4.152	0.678
Good relationship with studymates	-2.627	1.698	-5.964–0.711	0.123	Good relationship with studymates	3.802	2.091	-0.312–7.918	0.070
					Satisfaction with study choice	2.591	2.643	-2.609–7.792	0.328

*CI Confidence interval, *R2* = 0.246; ANOVA: F = 12.809, *p* = < 0.001

**CI Confidence interval,* R2* = 0.321; ANOVA: F = 13.737,* p* = < 0.0.001

## Discussion

This study explores health promoting resources, general health and wellbeing, and health promoting lifestyle factors among students in the fourth (T4) and final semesters (T6/7) in higher education in healthcare and social work programmes. The main results showed a relatively high SOC in the fourth semester, but it slightly decreased by the end of the education programme. A reason may be the increased demand for study performance. According to the Public Health Agency of Sweden [48], the opportunity to control and influence study time and the high workload are causing stress and making it difficult for students to relax. In a Norwegian study on stress and the degree of SOC among nursing students [49], a high SOC indicated less stress and better quality of life, while low SOC was associated with higher stress experiences and poorer quality of life. Thus, a high level of SOC can be seen as a resource for managing stress.

Although the students’ SOC slightly decreased as they approached the end of their education, they had better wellbeing in the final semester than in the fourth semester. This may have to do with their confidence in their own ability, for instance, how they can manage and understand different learning activities, examinations, and seminars. Research shows that there is a correlation between trust in one’s own abilities and health [50, 51]. According to Azizli et al. [50], planning creates feelings of control and of being prepared, which can lead to greater satisfaction with life. Duffy, Douglass and Austin [52] found that those students who had feelings of control and confidence in their career decisions had higher levels of academic satisfaction. The degree of self-efficacy is a personal characteristic and starts from the person’s subjective image of themselves [53]. Understanding the level of an individual’s self-efficacy could help prioritise health promotion efforts that provide the right conditions for increasing students’ SOC. Thus, teachers in higher education can strengthen students’ health promoting resources by creating a supportive environment.

Our results showed that fellow students and work in groups were more valued in the final semester. One can imagine that increased pressure in the final semester, with final examinations taking place, may result in low perceived wellbeing. Previous research suggests that access to social support during higher education studies can be important for the students´ wellbeing [27, 28, 54], especially in stressful situations [54]. Fjelkner-Phil [55] shows that multiplex networks, friendships, working and learning networks (i.e. group activity) overlap substantially, and are positively and significantly related to academic outcomes. Therefore, the friendship of fellow students and work in groups may be enough to maintain the SOC. Teachers may support students’ SOC by developing learning activities such as reflection activities or group work as a means of creating social interactions between students at the beginning of the education programme.

In our study, the meaningfulness and comprehensibility elements of the students’ SOC were relatively high, but manageability was low both in the fourth and final semesters. Previous research shows that a strong SOC can act as a buffer against long-term stress [43]. A study [56] focusing on the level of SOC among Swedish nursing staff indicates that nurses have difficulty managing stressful situations at work and suggests that a strong SOC is related to the ability to manage stress in working life. Another study [5] found that when nurses felt the work was meaningful, they perceived SOC. They felt togetherness and pride in their profession, even under conditions involving stress and a high workload. When our results showed that the students had relatively high meaningfulness and comprehensibility, this may indicate that they had resources to manage stress and felt the education meaningful and comprehended.

SOC makes it possible for people to comprehend their situation with the help of generalised and specific resistance resources against stress. This means that resistance resources enable the development of strong SOC [9, 10]. Extensive research on SOC provides evidence that a strong SOC both mediates and moderates stress [10], and thus helps meeting the challenges of an ever-changing world, not least in the context of care. It is therefore important to identify new *generalised resistance resources* (GRR) and *specific resistance resources* (SRR) that may be effective in a healthcare context [10, 57]. Teachers and supervisors should be aware of students’ needs during their studies to understand both their current situation and what their future professional role may entail. This may be done through targeted assignments, where the student reflects on a care situation and then shares their reflections with teachers and supervisors. The latter will then be able to assess how the student perceives the situation and respond pedagogically as well as support the student’s specific resistance resources. A way of managing stress and feeling that a situation is intelligible, manageable, and meaningful can be developed through support for salutogenic coping strategies and by prioritising health promotion efforts based on Antonovsky’s [9] salutogenic approach. Another way of managing stress and feeling that a situation is intelligible, manageable, and meaningful can be developed through a personal portfolio where the students can collect their reflections and receive support from teachers and supervisors [58]. It is important for the student to feel involved and to be able to influence the circumstances of their period of study, something that may then increase the degree of meaningfulness that is a prerequisite for both comprehensibility and manageability.

The results showed that high-intensity exercise was not a significant predictive factor for SHIS however it was found to be a significant predictive factor for general health in both the fourth and final semester. Also, high-intensity exercise as health promoting lifestyle factor showed a significant decrease between the fourth and final semesters. This decrease is problematic, seeing high-intensity exercise as health promoting lifestyle factor and predictive factor for good general health. The reasons for this decrease could be that the students in the final semester might be occupied with final examinations and producing their thesis. This is also in line with that there is an increase in sedentary lifestyles from semester four to the final semester, however this increase was not statistically significant. Another reason for this decrease in the final semester could be restrictions related to the COVID-19 pandemic that limited access to group/team training and high-intensity exercise. Other studies [58] have also found decreasing levels of physical activity during the pandemic. It is possible that students’ exercise during the pandemic could have shifted to moderate-intensity physical activities, which also are in line with our results, although not significant in this study.

Another possible COVID-19 effect on these results could be due to that the higher education programmes turned to distance learning during the period of data collection. Research has demonstrated a link between the COVID-19 pandemic and mental illness, poorer quality of life, and less life satisfaction [59–62]. An international study [63], in which students from 26 countries participated, found that depressive symptoms were more frequent among students in countries with strict measures regarding school closures and restrictions on movement outside the home. The restrictive measures had a significant impact on students’ everyday lives, with changes to social contacts as well as to teaching methods. However, the Nordic countries (and France) had the lowest levels of depressive symptoms among the countries represented in the study [63]. Persson et al. [64] suggest that the difference in this respect between students in Sweden and in many other countries may be accounted for by the fact that Sweden did not impose social lockdown, which may have had an impact on students’ mental health.

### Strengths and limitations

This study is based on longitudinal data from the research project “Health promoting factors in higher education for a sustainable working life” [44] and is a follow-up to the baseline study [37], which can be considered a strength in that it reduces the risk of publication bias. Another strength is that this study uses the SOC scale to examine/measure health promoting factors among young adults, which is uncommon in previous research.

Further, the risk of selection bias is small, as all students in the selected programmes were invited to participate in the study. However, there might be a risk of self-report bias, which may occur when the people filling out the questionnaire are the type of people who like to fill out questionnaires. A limitation is the low number of males. However, the majority of those who apply for these programs in Sweden are women [65], which makes the study population representative of the target group. A further limitation is that a considerable amount of students were lost to follow-up during the study period. Loss to follow-up has significant implications for longitudinal studies and can lead to biased estimates and limited generalizability. Another limitation of the study that must be considered is that its design does not lend itself to the calculation of effect, which is often a recommendation when statistical analyses are carried out. This study was conceived as a total population study, representing all students at six higher education programmes in healthcare and social work during their fourth and final semesters.

The longitudinal design allows for a two-year perspective, but as data were gathered cross-sectionally, it was not possible to establish cause-and-effect relationships. However, the study can provide an important overview of health, health promoting resources, and lifestyle factors reported by students in their fourth and final semesters.

## Conclusions

This study explores health promoting resources, general health and wellbeing, and health promoting lifestyle factors among fourth and final semester students in higher education in healthcare and social work. Students’ report of good general health, were associated with wellbeing, high-intensity physical training and no sleeping problems. However, only 6% of the students were reporting no sleeping problems. Furthermore, students in both the fourth and final semester reported relatively high SOC. SOC were also associated with good general health, wellbeing and no sleeping problems. Therefore, further research is needed with focus on promoting students’ ability to manage their lives and studies for better health and wellbeing, particularly according to sleeping quality during, as well as after, their education.

## Electronic supplementary material

Below is the link to the electronic supplementary material.

Supplementary Material 1

## Data Availability

The datasets and materials used and analysed during the current study are available from the corresponding author upon reasonable request.

## Abbreviations

QPS Nordic: The General Nordic Questionnaire
SHIS: Salutogenic Health Indicator Scale
SOC: Sense of Coherence
