# Supplementary material for: Health promoting resources and lifestyle factors among higher education students in healthcare and social work programmes: a survey with a longitudinal multicentre design
Source: BMC Public Health. 2024 Nov 8;24:3097. doi: 10.1186/s12889-024-20506-9 (PMC11545803; doi:10.1186/s12889-024-20506-9)
Supplement: Supplementary file 1 — Supplementary Material 1 [file 12889_2024_20506_MOESM1_ESM.docx]

| Supplementary table. Univariate logistic regression analysis of predictive factors of general health and univariate linear regression analysis of predictive factors of SOC and SHIS in the fourth and last semester | | | | | | | | | | |  |
| --- | --- | --- | --- | --- | --- | --- | --- | --- | --- | --- | --- |
|  | | | T4 | |  | T6/7 | | | | | |
| *Good General health^b^* | B (SE) | Exp(B) | | *p-*value | | B (SE) | Exp(B) | *p-value* | | |  |
| Perceived good wellbeing | 2.79 (0.316) | 16.435 | | **<0.001** | | 2.489 (0.353) | 12.047 | | **<0.001** |  |  |
| High-intensity exercise (Physical exercises) > 60-90 min/week | 1.661 (0.321) | 5.267 | | **<0.001** | | 1.035 (0.355) | 2.814 | | **0.004** |  |  |
| Moderate-intensity physical activity > 150 min/week | 0.529 (0.299) | 1.697 | | 0.077 | | 0.330 (0.319) | 1.391 | | 0.302 |  |  |
| Sedentary <10 h/day | 0.950 (0.308) | 2.585 | | **0.002** | | -0.028 (0.384) | 0.972 | | 0.941 |  |  |
| No Sleeping problems | 1.451 (0.416) | 0.234 | | **<0.001** | | 2.069 (0.481) | 0.126 | | **<0.001** |  |  |
| Daily intake of vegetables | -0.541 (0.318) | 0.582 | | 0.089 | | -0.506 (0.351) | 0.603 | | 0.149 |  |  |
| No Consumption of alcohol | 0.188 (0.280) | 1.207 | | 0.502 | | 0.49 (0.318) | 1.050 | | 0.878 |  |  |
| No Smoking | 0.200 (0.464) | 1.221 | | 0.667 | | -0.137 (0.637) | 0.872 | | 0.830 |  |  |
| No Daily snuff | 0.525 (0.318) | 1.691 | | 0.099 | | 0.347 (0.377) | 1.414 | | 0.358 |  |  |
| Support from studymates | 0.819 (0.294) | 2.267 | | **0.005** | | 0.738 (0.336) | 2.092 | | **0.028** |  |  |
| Studymates listening | 0.770 (0.310) | 2.160 | | **0.013** | | 0.824 (0.350) | 2.280 | | **0.018** |  |  |
| Satisfaction with the study choice | 0.917 (0.493) | 2.642 | | **0.049** | | 1.686 (0.566) | 5.400 | | **0.003** |  |  |
| Satisfaction with the studies | 1.357 (0.454) | 3.885 | | **0.003** | | -2.052 (0.503) | 0.128 | | **<0.001** |  |  |
| Talk to friends about the studies | 0.893 (0.318) | 2.442 | | **0.005** | | 1.007 (0.372) | 2.736 | | **0.007** |  |  |
| Good relationship with studymates | 1.258 (0.353) | 3.518 | | **<0.001** | | 0.903 (0.471) | 2.468 | | 0.055 |  |  |
| Values group work | 0.207 (0.328) | 1.230 | | 0.528 | | 0.925 (0.369) | 2.521 | | **0.012** |  |  |
| *SOC^a^* | β | SE (β) | | *p-value* | | β | SE (β) | | *p-value* |  |  |
| General good health | 12.164 | 1.597 | | **<0.001** | | 8.935 | 1.886 | | **<0.001** |  |  |
| Perceived good wellbeing | 15.456 | 1.362 | | **<0.001** | | 13.625 | 1.476 | | **<0.001** |  |  |
| High-intensity exercise (Physical exercises) > 60-90 min/week | 2.736 | 1.094 | | **0.013** | | 1.020 | 1.335 | | 0.442 |  |  |
| Moderate-intensity physical activity > 150 min/week | 2.793 | 1.097 | | **0.011** | | 2.823 | 1.319 | | **0.033** |  |  |
| Sedentary <10 h/day | 5.246 | 1.418 | | **<0.001** | | 4.691 | 1.584 | | **0.003** |  |  |
| No Sleeping problems | 13.063 | 2.217 | | **<0.001** | | 13.633 | 2.675 | | **<0.001** |  |  |
| Daily intake of vegetables | 4.204 | 1.375 | | **0.002** | | -4.140 | 1.581 | | **0.009** |  |  |
| No consumption of alcohol | -0.613 | 1.111 | | 0.581 | | -1.892 | 1.332 | | 0.156 |  |  |
| No Smoking | 1.308 | 1.916 | | 0.495 | | -1.009 | 2.656 | | 0.704 |  |  |
| No Daily snuff | -1.463 | 1.381 | | 0.290 | | -1.715 | 1.664 | | 0.303 |  |  |
| Support from studymates | 6.162 | 1.275 | | **<0.001** | | 6.011 | 1.532 | | **<0.001** |  |  |
| Studymates listening | 7.546 | 1.365 | | **<0.001** | | 5.494 | 1.639 | | **<0.001** |  |  |
| Satisfaction with the study choice | 6.287 | 2.548 | | **0.014** | | 11.150 | 3.355 | | **<0.001** |  |  |
| Satisfaction with the studies | 5.044 | 2.454 | | **0.040** | | 10.597 | 3.029 | | **<0.001** |  |  |
| Talk to friends about the studies | 6.993 | 1.467 | | **<0.001** | | 7.434 | 1.865 | | **<0.001** |  |  |
| Good relationship with studymates | 8.199 | 1.774 | | **<0.001** | | 6.158 | 2.487 | | **<0.001** |  |  |
| Values group work | -0.596 | 4.300 | | 0.890 | | 6.275 | 1.788 | | **<0.001** |  |  |
|  |  |  | |  | |  |  | |  |  |  |
| SHIS | β | SE (β) | | *p*-value | | β | SE (β) | | *p*-value |  |  |
| General good health | 9.012 | 1.369 | | **<0.001** | | 9.420 | 1.570 | | **<0.001** |  |  |
| Perceived good wellbeing | 13.036 | 1.150 | | **<0.001** | | 12.552 | 1.204 | | **<0.001** |  |  |
| High-intensity exercise (Physical exercises) > 60-90 min/week | 2.632 | 0.913 | | **0.004** | | 2.574 | 1.117 | | **0.022** |  |  |
| Moderate-intensity physical activity > 150 min/week | 1.139 | 0.923 | | 0.218 | | 0.859 | 1.120 | | 0.444 |  |  |
| Sedentary >10 h/day | 3.788 | 1.183 | | **0.001** | | 2.365 | 1.351 | | 0.081 |  |  |
| No Sleeping problems | 9.811 | 1.907 | | **<0.001** | | 12.537 | 2.272 | | **<0.001** |  |  |
| Daily intake of vegetables | -3.067 | 1.151 | | **0.008** | |  |  | |  |  |  |
| No Consumption of alcohol | -1.165 | 0.925 | | 0.209 | | -0.737 | 1.126 | | 0.513 |  |  |
| No Smoking | 1.936 | 1.601 | | 0.227 | | 2.529 | 2.140 | | 0.238 |  |  |
| No Daily snuff | -0.070 | 1.150 | | 0.952 | | -0.563 | 1.432 | | 0.694 |  |  |
|  |  |  | |  | |  |  | |  |  |  |
| Support from studymates | 3.186 | 1.080 | | **0.003** | | 4.454 | 1.298 | | **<0.001** |  |  |
| Studymates listening | 3.664 | 1.167 | | **0.002** | | 3.934 | 1.408 | | **0.005** |  |  |
| Satisfaction with the study choice | 4.092 | 2.099 | | 0.052 | | 12.122 | 2.743 | | **<0.001** |  |  |
| Satisfaction with the studies | 6.322 | 2.131 | | **0.003** | | 13.770 | 2.456 | | **<0.001** |  |  |
| Talk to friends about the studies | 3.768 | 1.239 | | **0.002** | | 5.593 | 1.550 | | **<0.001** |  |  |
| Good relationship with studymates | 3.463 | 1.523 | | **0.023** | | 7.790 | 2.026 | | **<0.001** |  |  |
| Values group work | 2.744 | 1.118 | | **0.014** | | 3.642 | 1.541 | | **0.019** |  |  |
|  |  |  | |  | |  |  | |  |  |  |
